# Prevalence and Risk Factors of Hypertension in Two Communes in the Vietnam Northern Mountainous, 2017

**DOI:** 10.1155/2018/7814195

**Published:** 2018-10-09

**Authors:** Nhon Bui Van, Quyet Pham Van, Long Vo Hoang, Tung Bui Van, Nguyen Nguyen Hoang, Khanh Do Nam, Dinh-Toi Chu

**Affiliations:** ^1^Hanoi Medical University, Hanoi 100000, Vietnam; ^2^Faculty of Biology, Hanoi National University of Education, Hanoi 100000, Vietnam; ^3^Institute for Research and Development, Duy Tan University, Da Nang 550000, Vietnam

## Abstract

**Background:**

The aims were to characterize the prevalence of hypertension (HTN) and explore its associations in the northern mountainous.

**Methods:**

We carried out a cross-sectional study in two communes in Chiem Hoa district, Tuyen Quang province, between June and November 2017. All subjects at the age of 18 years and over currently living in two communes. The usage of the descriptive statistics was to characterize the HTN prevalence. We used the univariate and multivariate models of logistic regression to determine the prevalence and related factors of HTN.

**Results:**

There were 319 people with overall HTN in the total of 675 participants. Among people with HTN, there were 101 ones with isolated systolic hypertension (ISH). The proportion of HTN among the Tay ethnic group was 47.6%. The factors related to HTN included group, body mass index (BMI), low physical fitness, and waist-hip ratio (WHR). These factors as well as the ethnicity were significantly associated with ISH.

**Conclusions:**

Two communes of Chiem Hoa district in Tuyen Quang province had a high prevalence of HTN. Age, BMI, WHR, and physical activity were the risk factors of overall HTN and ISH. In particular, ISH was affected by ethnicity.

## 1. Introduction

Hypertension (HTN) is not only known as a risk factor contributing to cardiovascular disease (CVD) but also a health issue in the community [1]. The global population currently suffering from HTN are estimated to be more than 1.5 billion people [1–3]. Approximately 7.6 million early deaths were caused to HTN, which was about 13.5% of the global total [4]. Although the proportion of HTN is increasing in the developed countries [5], many low and middle-income countries (LMICs) see a faster rise in the proportion of HTN [6]. Isolated systolic hypertension (ISH) determined as blood pressure (BP) ≥140/<90 mmHg is common in the elderly, besides it can also be found in the young [7–10]. The risk factors for HTN are known to be age, low physical fitness, tobacco usage, unhealthy eating, and high salt consumption [11].

HTN is known as a significant contributing factor for the overall burden of illness in developing countries in Southeast Asia including Vietnam [12, 13]. Vietnam is experiencing the double burden of old transmissible diseases as well as emerging noncommunicable diseases [14]. A quarter of Vietnamese people aged 25 and older, which was equivalent to approximately 11 million populations, suffered from HTN [15]. The previous studies indicated that the prevalence of HTN was increasing in rural communities in Vietnam, particularly HTN prevalence among people residing in urban areas was more than twice that of those in rural areas [15, 16]. Compared to regions belonging coastal and highland zone, the prevalence of HTN in cities and lowlands was higher because of the strong industrialization and modernization worldwide in recent decades [15].

Kim Binh and Xuan Quang are two low economic communes in Tuyen Quang district of Vietnam, with limited infrastructure and difficult access to health services. Particularly, these areas are inhabited by the ethnic minorities, mainly the Tay ethnic group, accounting for 25.6% of the population, the Dao ethnic group accounts for 13.3% of the population, and the Nung ethnic group accounts for 2.1% of the population [17]. Studies in Vietnam have focused only on HTN in different geographical and socioeconomic settings [15, 16, 18]. No studies have currently focused on HTN prevalence in ethnic minorities in the northern mountainous area and therefore the information on HTN in these areas is not available. For that reason, this study with the aim is to evaluate HTN prevalence and its associated factors in two communes in the northern mountainous area of Vietnam.

## 2. Methods

### 2.1. Study Design and Setting

We carried out this study in two communes (Kim Binh and Xuan Quang) in Chiem Hoa district, Tuyen Quang province. A cross-sectional descriptive design was used. People at the age of 18 years or above residing in these two communes were enrolled.

### 2.2. Sample Size

The sample size was calculated as follows:

(1) $$\begin{array}{c} n=Z_{\left(1-\alpha /2\right)}^{2}\times \left(\;\frac{1-p}{\varepsilon^{2}\times p}\;\right) \end{array}$$

n is sample size. *α* is level of statistical significance (choose *α* = 0.05 with 95% confidence level then replace the table that is *Z*_(1−*α*/2)_^2^ = 1.96). p = 0.244 which was recorded prevalence of HTN in the mountainous population [19]. *ε* = 0.1, desired error between study sample and population. The research sample size was calculated as 607. Approximately 15% of the subjects may be the possibility of refusals and/or losses. Therefore, a total of 675 subjects in two communes were involved in the study.

### 2.3. Samplings

The usage of systematic random sampling was to pick study subjects living in these two communes, including three steps: (i) to make a list of all people in 2 communes, including Kim Binh and Xuan Quang; (ii) to determine the sample interval K = 5; (iii) to select the first object randomly between 1 and 5 and then the next object was chosen by the extra distance until there are enough 675 objects.

### 2.4. Measurements

The Omron HBP-1300 BP Monitor was used for measurement of BP. BP was recorded 3 times on the right arm. Before the measurement, all participants took a rest within 5 minutes. An interval was 30 sec between two consecutive measurements. When subjects were ready to take BP, they were required to keep calm and not think about stressful things. Then, subjects were required to take of excess clothing because they might impede the BP monitor or limit blood flow and then sit comfortably for five minutes with the back rested on a chair, as well as the legs and ankles of subject uncrossed. The average of systolic blood pressure (SBP) and diastolic blood pressure (DBP) of the three measurements were calculated. Before taking your BP, the subjects was suggested that the consumption of a caffeinated beverage or smoking and physical activities were not allowed 30 minutes before BP checking. Body weight and height were measured by weight and height scale without heavy clothing and shoes, respectively. Measurement of waist circumference (WC) was carried out by a tape measure which was put at the approximate midpoint between the top of the pelvis and the below margin of the last rib. Taking around the largest part of your hips, the widest part of your buttocks was conducted to measure hip circumference (HC).

### 2.5. Definitions

BP was classified according to the JNC 7 guidelines [20]. HTN was specified that SBP was 140 mmHg or higher and/or DBP was 90 mmHg or higher, if the medications used to treat HTN were used by the individuals for 2 weeks. ISH having a SBP ≥140 mmHg and DBP <90 mmHg was used to diagnose. The calculation for body mass index (BMI) was by taking kg/m2 for each individual. According to BMI recommendation for Asian people, underweight was less than 18.5, normal weight was from 18.5 to 23, overweight was a range of 23 to 25, and obesity was 25 or over [21]. Waist-to-hip ratio (WHR) measured the ratio of your WC to your HC. Subjects smoking at least one cigarette per day within 6 months or more were considered as cigarette smokers. The definition of alcohol usage was as drinking at least once a week during a year ago.

### 2.6. Data Analysis

The data was analyzed by taking use of STATA 12.0 software. The significance level was considered at p<0.05. To describe the status of HTN in 2 communes, the descriptive method (such as percentage and mean) was applied. The univariate and multivariate logistic regression was used to determine related factors to HTN.

### 2.7. Research Ethics

All subjects were explained about the procedure and the purpose of the study. Subjects participated in the study under a voluntary basis. Referral services were provided if required at the commune health centres. Personal information of the subjects was kept confidential and encrypted. People with newly detected HTN are consulted and treated.

## 3. Results

Of 675 subjects, about 67.8% of them were female. Tay ethnic group accounted for 70.7%, while the figure for Kinh ethnic group and other ethnic groups was significantly lower, at 14.5% and 14.8%, respectively. The median age of subjects was 60, the youngest was 18, and the oldest was 95. The age group with highest proportion was ≥55 years (64.7%), and the age group accounted for the lowest (8.7%). Most people worked in agriculture (90.4%); the rest were in other occupations (9.6%). People with normal weight were 58.8% (Table 1).

Table 2 depicts the HTN prevalence among the ethnic groups according to BP category. The overall HTN prevalence among all subjects was found to be 47.3%. The figures for HTN among ethnic groups were nearly 50%. With regard to HTN, the figure for Tay ethnic group was 47.6%. The overall prevalence of ISH was 31.7%. The figure for ISH among Tay ethnic group was the highest, with 37.9%.

The proportion of HTN in men was higher than that in women (OR^1^ = 1.61, 95% CI: 1.16–2.23). The percentage of HTN in the age group of ≥55 and the age group of 35–54 group was higher than that in the age group of 18–34 (≥55: OR^1^ = 7.86, 95% CI: 3.65–16.91; OR^2^ = 5.82, 95% CI: 2.70–12.55; and 35–54: OR^1^ = 2.40, 95% CI: 1.09–5.28; OR^2^ = 2.40, 95% CI: 1.08–5.34). HTN in the obese group (BMI ≥25.0) was higher than that in the normal group (18.5 ≤ BMI <23.0) (OR^1^ = 1.99, 95% CI: 1.25–3.19). The prevalence of HTN in abdominal obesity subjects (WHR ≥0.95) was higher than that of normal subjects (WHR <0.85) (OR^1^ = 5.17, 95%CI: 2.94–9.08; OR^2^ = 2.61, 95% CI: 1.44–4.72). The prevalence of HTN in the insufficient physical activity group was higher than in the sufficient physical activity group (OR^1^ = 2.24, 95% CI: 1.63–3.08; OR^2^ = 1.65, 95% CI: 1.16–2.36) (Table 3).

The percentage of ISH was higher among people aged ≥55 than among people aged 35–54 (OR^1^ = 5.03, 95% CI: 2.44–10.38; OR^2^ = 4.81, 95% CI: 2.27–10.21). The proportion of ISH was higher in Tay ethnic group than in Kinh ethnic group (OR^1^ = 3.37, 95% CI: 1.41–8.01; OR^2^ = 4.27, 95% CI: 1.77–10.32). The proportion of ISH was higher among people with abdominal obesity than others with in the nonabdominal obesity (0.85≤ WHR <0.9: OR^1^ = 2.35, 95% CI: 1.26–4.38; 0,9≤ WHR <0,95: OR^1^ = 3.30, 95%CI: 1.74 – 6.27; WHR ≥0,95: OR^1^ = 3.75, 95% CI: 1.87–7.51). The proportion of ISH in the insufficient physical activity group was significantly higher than that in the sufficient physical activity group; the odds ratio was statistically significant in the univariate analysis (OR^1^ = 1.84, 95% CI: 1.17–2.89) (Table 4).

## 4. Discussion

Many studies have reported that the rate of HTN and CVD in Southeast Asia region continues to increase. HTN has been showed as one of the most common CVD in this area, and the HTN prevalence in different districts and countries differs dramatically. The prevalence of HTN in two mountainous communes in our study was 47.3% (319/675 people), much higher than the overall prevalence in the northern mountainous provinces of Vietnam in the study of Pham Gia Khai et al. (16.3%) [22]. This result shows also significantly higher prevalence of HTN than in previous studies in Vietnam, 11.2% in 1992 [23], 16.3% in 2001 [24], 18.3% in 2005 [25], and 25.1% from 2002 to 2008 [15]. The proportion of HTN in these two communes is completely consistent with the result of Nguyen Lan Viet in a report on the overall HTN prevalence in the population of Vietnam. There were 2577 of 5454 people with HTN (47.3% in 2016) [26]. Compared with the proportion of HTN in some countries in the Southeast Asia such as 22% in Thailand [27], 32.2% in Indonesia [28], and 22% in Myanmar [29], the figure for HTN in our study is much higher. Our result is similar to data from Armenia (47.8%) and Botswana (47.5%) [6]. It might be because Armenia and Botswana worldwide belong to LMICs as well as one of the thirty developing countries where are entirely surrounded by land [30]. Thus, people are not fully aware of the dangerous risks of HTN and access to healthcare services for HTN diagnosis and prevention is limited. As well as in two mountainous communes of Vietnam, this is an area with many socioeconomic difficulties in taking approach to health services.

A report of the Vietnam Committee on Ethnic Minority Affairs showed that there were differences among the ethnic groups in Vietnam. Particularly Vietnam's 53 ethnic minorities are scattered all over the country. There are ethnic groups with populations of more than one million people and groups of several hundred to under 5000 people. In addition, ethnic groups have a large gap in life expectancy and child mortality. HTN and ethnicity are the health-related issues that increasingly paid attention in Vietnam. With regard to HTN, the figure for ethnic groups was found to be nearly 50%. Particularly, our study found that the prevalence of HTN among Tay ethnic group (47.6%) was considerably high compared to figure for Tay ethnic group in Bac Can province in 2009 (15%) [31]. Tay ethnic group in present study was also significantly higher prevalence of HTN than other ethnic groups in previous studies in Vietnam, 26.7% among the Ede [32], 19.2% among Thai [33], 38.9% among Khmer [34], 18.7% among Nung [35], and 2.20% among K'Ho [36]. The difference of prevalence observed between the present study and other studies could be due to social and cultural differences, changes in dietary and lifestyle, an increase in life expectation, and also the age as well as the research methodology used. Moreover, the overall prevalence of ISH was 31.7%. This figure for among Tay ethnic group was the highest, with 37.9%. This is a remarkable prevalence to have suitable intervention policies for HTN among Tay ethnic group in two communes of study.

The prevalence of HTN in the 35–55 age group and age group over 55 was 2.40 times higher (95% CI: 1.08–5.34) and 5.82 times (95% CI: 2.70–12.55) than that in the 18–34 age group (multivariate analysis). This result was consistent with the study by Pham Gia Khai [22], Nguyen Thi Kim Hoa (2009) in Huong Thuy district, Thua Thien Hue province [37]. It be might because the age was an integral part of general CVD and HTN in particular [38].

With respect to the risk of HTN in obese and overweight people, our study indicated that the HTN risk in obesity subjects (BMI ≥25) was 1.99 times higher (95% CI: 1.25–3.19) than that in normal subjects (18.5≤ BMI <23). These results were consistent with the previous studies in Vietnam [19, 39]. In addition, an association between WHR and HTN was found in our study. People with male waist obesity (WHR ≥0.95) had 2.61 times (95% CI: 1.44–4.72) higher risk of HTN than those of WHR <0.85. People with WHR in the range of 0.9 to 0.95 had a 2.3-fold (95% CI: 1.52–3.61) increased risk of HTN than people with a WHR <0.85. Our results were consistent with the study of Pham Gia Khai et al. (2003) where the risk for HTN in people with WHR ≥0.95 was 3.00 times higher than in those with the WHR <0.85. This finding indicated that the importance of weight control was in reversing the risk of HTN.

Our results showed that insufficient physical activity was a risk factor for HTN. People who were not physically active have a 1.65 times (95% CI: 1.16–2.36) higher risk of HTN compared to people with physical activity. This result was similar to the study of Hoang Van Ngoan conducted in Huong Thuy district, Thua Thien Hue [40]. It was estimated that lack of physical activity accounted for 5 to 13% of the risk for developing HTN [23].

Our study also found a link between ISH and some risk factors. Patients over 55 years of age were at higher risk of ISH (univariate OR: 5.03, 95% CI: 2.44–10.38; multivariate OR: 4.81, 95% CI: 2.27–10.21). In addition, subjects with WHR of 0.85 to 0.90, 0.90 to 0.95 and ≥0.95 were at 2.35, 3.30 and 3.75 times higher risk of ISH than those with WHR <0.85 (univariate analysis). Therefore, ISH is a major burden for people over 55 years. In our study, the Tay ethnic group were at higher risk of ISH than the Kinh ethnic group (univariate OR: 3.37, 95% CI: 1.41–8.01; multivariate OR: 4.27, 95% CI: 1.77) % CI 1.77–10.32). It might be explained that the access to health information and health services of the Tay ethnic group is limited so the prevention of risk factors for ISH has not been paid much attention. Furthermore, these are two poor communes in the mountainous of Northern Vietnam, which is more complex topographic feature than other areas in the Northern Vietnam. Hence, the majority of Tay people have difficulties in receiving information via several communal health workers. The majority of households in this area do not own computers, Internet, and smartphones to approach information and knowledge of health. Particularly these communes are mainly Tay ethnic group in our study. Compared to other ethnic groups, people among Tay ethnic group is more concentrated in the mountainous areas, so they have specific habits of culture and customs different from other ethnic groups, particularly this is daily bad routines. In this study, the low physical activity is also one of the contributing factors to ISH. Compared with the sufficient physical activity group, the insufficient physical activity group had a 1.84-fold increase in ISH (95% CI: 1.17 – 2.89) (multivariate analysis).

This study used a cross-sectional design which does not help determine any causal relations between risk factors and the development of HTN. Research with the appropriate design is required to explore if any cause-and-effect relationship exists. Another limitation of this study was the inability to assess the lifestyle and sociocultural aspects between ethnic groups in this area. Future study needs to measure metabolic syndrome including blood sugar and cholesterol because metabolic syndrome includes five risk factors that grow the probability of developing CVD. In this study, we did not use any longitudinal measurements. As this was a cross-sectional measurement and BP is known to fluctuate.

## 5. Conclusion

A remarkably high overall prevalence of HTN in two mountainous communes including Kim Binh and Xuan Quang in Tuyen Quang province was found. The prevalence of HTN among Tay ethnic group was considerably higher than the figures in previous studies among other ethnic groups in Vietnam. The significant associations of age, BMI, WHR, and physical activity with HTN were found. Particularly ISH is also affected by ethnicity.

## Figures and Tables

**Table 1:** Demographic characteristics of research subjects (*n* = 675).

Characteristics	No.	%
Age groups		
18–34	59	8.7
35–54	179	26.5
≥55	437	64.7
Median (Min – Max )	60 ( 18 – 95)	

Gender		
Male	217	32.2
Female	458	67.8

Ethnic groups		
Kinh	98	14.5
Tay	477	70.7
Dao	57	8.4
Others^a^	43	6.4

Occupation		
Farmer	610	90.4
Others^b^	65	9.6

Educational		
No school / illiteracy	61	9.1
Primary	254	37.6
Junior high school	266	39.4
High school or higher	94	13.9

BMI		
Underweight (BMI <18.5)	97	14.4
Normal (18.5≤ BMI <23.0)	395	58.5
Overweight (23.0≤ BMI <25.0)	90	13.3
Obese (BMI ≥25.0)	93	13.8

^*a*^ *Others: Nung, Mong, and Muong ethnic group.*

^*b*^ *Others: officers, manual worker, retirees, and freelancer.*

**Table 2:** The prevalence of hypertension of the ethnic groups according to blood pressure category (*n* = 675).

BP category (mmHg)	Kinh		Tay		Dao		Others		Total	
	n	%	n	%	n	%	n	%	n	%
Optimal	31	31.6	107	22.4	16	28.1	11	25.6	165	24.4

Normal	8	8.2	73	15.3	5	8.8	4	9.3	90	13.3

High normal	14	14.3	70	14.7	10	17.5	7	16.3	101	15.0

HTN	45	45.9	227	47.6	26	45.6	21	48.8	319	47.3

Total	98	100	477	100	57	100	43	100	675	100

ISH (n=319)	6	13.3	86	37.9	5	19.2	4	19.0	101	31.7

**Table 3:** Related factors of hypertension (*n* = 675).

Related factors	Univariate			Multivariate		
	OR^1^	95%CI		OR^2^	95%CI	
Gender (male vs. female)	1.61^*∗*^	1.16	2.23	1.57	0.91	2.71

Age groups (vs. 18–34)						
35–54	2.40^*∗*^	1.09	5.28	2.40^*∗*^	1.08	5.34
≥55	7.86^*∗*^	3.65	16.91	5.82^*∗*^	2.70	12.55

Ethnic groups (vs. Kinh)						
Tay	1.07	0.69	1.65	1.15	0.72	1.85
Dao	0.99	0.51	1.91	1.06	0.53	2.14
Others	1.12	0.55	2.31	1.24	0.56	2.76

BMI^a^ (vs. 18.5≤ BMI <23.0)						
BMI <18.5	0.84	0.54	1.33	0.76	0.46	1.27
23.0≤ BMI <25.0	1.06	0.67	1.67	1.08	0.64	1.79
BMI ≥25.0	1.99^*∗*^	1.25	3.19	1.54	0.91	2.62

WHR^b^ (vs. WHR <0.85)						
0.85≤ WHR <0.90	1.39	0.95	2.06	1.02	0.66	1.55
0.90≤ WHR <0.95	2.34^*∗*^	1.52	3.61	1.37	0.84	2.22
WHR ≥0.95	5.17^*∗*^	2.94	9.08	2.61^*∗*^	1.44	4.72

Salty diet (vs. No)						
Yes	0.95	0.63	1.41	1.01	0.64	1.56

Physical activity (vs. Yes)						
No	2.24^*∗*^	1.63	3.08	1.65^*∗*^	1.16	2.36

Smoking (vs. No)						
Yes	1.09	0.73	1.64	0.67	0.37	1.21

Drinking alcohol (vs. No)						
Yes	1.41	0.96	2.06	1.13	0.64	1.98

^*∗*^ *p* < 0.05

^1^ *Univariate*. ^2^*Multivariate*.

^*a*^ *BMI: body mass index.*

^*b*^ *WHR: waist-hip ratio.*

**Table 4:** Related factors of isolated systolic hypertension (*n* = 675).

Related factors	Univariate			Multivariate		
	OR^1^	95%CI		OR^2^	95%CI	
Gender (Male vs. Female)	1.08	0.69	1.69	0.74	0.36	1.51

Age groups (vs. 35–54)						
18–54	-	-	-	-	-	-
≥55	5.03^*∗*^	2.44	10.38	4.81^*∗*^	2.27	10.21

Ethnic groups (vs. Kinh)						
Tay	3.37^*∗*^	1.41	8.01	4.27^*∗*^	1.77	10.32
Dao	1.47	0.43	5.09	1.93	0.54	6.87
Others	1.57	0.42	5.92	2.53	0.64	9.97

BMI^a^ (vs. 18.5≤ BMI <23.0)						
BMI <18.5	0.71	0.35	1.45	0.85	0.39	1.84
23.0≤ BMI <25.0	1.65	0.92	2.96	1.86	0.98	3.55
BMI ≥25	1.38	0.75	2.52	1.16	0.60	2.23

WHR^b^ (vs. WHR <0.85)						
0.85≤ WHR <0.9	2.35^*∗*^	1.26	4.38	1.68	0.86	3.24
0.90≤ WHR <0.95	3.30^*∗*^	1.74	6.27	1.94	0.96	3.94
WHR ≥0.95	3.75^*∗*^	1.87	7.51	1.68	0.78	3.63

Salty diet (vs. No)						
Yes	0.74	0.40	1.35	0.82	0.43	1.57

Physical activity (vs. Yes)						
No	1.84^*∗*^	1.17	2.89	1.33	0.80	2.19

Smoking (vs. No)						
Yes	0.91	0.51	1.63	0.87	0.40	1.90

Drinking alcohol (vs. No)						
Yes	1.05	0.63	1.79	1.26	0.61	2.61

^*∗*^ *p* < 0.05

^1^ *Univariate*. ^2^*Multivariate*.

^*a*^ *BMI: body mass index.*

^*b*^ *WHR: waist-hip ratio.*

## Data Availability

The data used to support the findings of this study are available from the corresponding author upon request.
